# Racial/Ethnic Disparities in Lung Cancer Surgery Outcomes in the USA

**DOI:** 10.3390/epidemiologia6020018

**Published:** 2025-04-11

**Authors:** Ivana Vasic, Kian C. Banks, Julia Wei, Leyda Marrero Morales, Zeuz A. Islas, Nathan J. Alcasid, Cynthia Susai, Angela Sun, Katemanee Burapachaisri, Ashish R. Patel, Simon K. Ashiku, Jeffrey B. Velotta

**Affiliations:** 1 Division of Thoracic Surgery, Kaiser Permanente Oakland Medical Center, 3600 Broadway, Oakland, CA 94611, USA; ivasic@alamedahealthsystem.org (I.V.); nalcasic@alamedahealthsystem.org (N.J.A.); csusai@alamedahealthsystem.org (C.S.); ashish.r.patel@kp.org (A.R.P.); jeffrey.b.velotta@kp.org (J.B.V.); 2 Department of Surgery, University of California, San Francisco East Bay, 1411 E 31st St, Oakland, CA 94602, USA; 3 Division of Research, Kaiser Permanente Northern California, 2000 Broadway, Oakland, CA 94612, USA; 4 School of Medicine, University of California, San Francisco, 533 Parnassus Ave, San Francisco, CA 94143, USA; leyda.morales@ucsf.edu (L.M.M.); katemanee.burapachaisri@ucsf.edu (K.B.); 5 Kaiser Permanente Bernard J. Tyson School of Medicine, 98 S Los Robles Ave, Pasadena, CA 91101, USA; zeuz.islas@kp.org

**Keywords:** racial/ethnic disparities, integrated health system, lung resection, ED return, outpatient visits

## Abstract

**Background/Objectives**: Sparse data exist identifying racial/ethnic outcome disparities among patients with lung cancer, specifically regarding healthcare utilization patterns, such as emergency department visits and outpatient follow-ups. We aimed to utilize our large, multicenter, and ethnically diverse integrated health system to assess for such disparities among patients undergoing pulmonary resections for lung cancer. **Methods**: The cohort comprised all patients undergoing pulmonary resections for lung cancer at our integrated health system from 1 January 2016 to 31 December 2020. Outcomes including the length of stay (LOS), 30-day return to the emergency department (30d-ED), 30-day readmission, 30- and 90-day outpatient appointments, and 30- and 90-day overall mortality were compared by race/ethnicity. Multivariable logistic and linear models adjusted for age, sex, body mass index (BMI), Charlson Comorbidity Index scores, procedure approach, neighborhood deprivation index (NDI), cancer stage, receipt of adjuvant chemotherapy, and insurance. **Results**: Of the 645 included patients, non-Hispanic White patients tended to be older and live in the least deprived neighborhoods. Among each race/ethnicity, the percentage of patients insured by Medicaid was highest among Asian patients. On bivariate analysis, only the outcome of surgical outpatient appointments within 30 days had differing distributions by race/ethnicity with no other significant associations between race/ethnicity and other outcomes; however, multivariable analysis showed Asian patients having lower odds of 30d-ED (adjusted odds ratio 0.51; 95% CI 0.27–0.98) while those with Medicaid insurance had higher odds of 30d-ED (adjusted odds ratio 3.29; 95% CI 1.26–8.59). **Conclusions**: Despite parity across clinical outcomes, some patient encounter-related differences still exist within our system. To better understand racial/ethnic disparities in care, systems must track such disparities in addition to clinical outcomes.

## 1. Introduction

Current research is increasingly focusing on racial and ethnic disparities in healthcare, providing medical communities with a better understanding of how these disparities impact health outcomes. The Center for Disease Control health report prior to the COVID-19 pandemic demonstrated disturbing differences in life expectancy among different racial groups: life expectancy for the Black population was 74.7 years compared with 78.6 years for the White population [1]. This report also highlighted the overall mortality rate of various ethnic groups across different health conditions.

Disparities in cancer incidence and outcomes are complex, stemming from a mix of genetics, social determinants of health, and behavioral factors. Despite progress in developing frameworks that consider both biological and social aspects of cancer, inequities in health outcomes persist. Lung cancer remains the leading cause of cancer-related death in the United States, with the highest overall mortality among African American men [1,2]. Lung cancer portrays the greatest socioeconomic disparities amongst all cancers [3]. In addition, factors such as age at diagnosis, smoking status, and disease stage at diagnosis vary among ethnic groups and may further play a role in cancer outcomes [1,2]. While there is considerable research describing the risk factors for incidence among different ethnic groups, there are limited data on disparities in outcomes. Studying health outcomes in a US healthcare system presents its own challenges. Despite the implementation of the Affordable Care Act, millions of Americans remain uninsured [4].

A large systemic review indicated that even factors like where cancer was diagnosed (emergency room vs. outpatient appointment) influenced patients’ healthcare experiences and their trust in providers [5]. A retrospective cohort study of over 2000 lung cancer patients showed that when compared with non-Hispanic White patients, African American and Asian patients experienced a lower ability to get care and a lower ability to get care quickly, respectively [6]. Patient interaction within healthcare systems is thus a crucial aspect of care with significant short- and long-term effects on patient health outcomes [7,8]. To understand disparities, more granular data need to be collected. Studying factors such as access to care, quality of care, and patients’ preferences can help us identify and address disparities in healthcare outcomes.

Our goal was to utilize a large diverse integrated health system and study racial/ethnic outcome disparities among patients undergoing pulmonary resection for primary lung cancer. We primarily focused on the hospital length of stay (LOS), hospital readmission within 30 and 90 days, ICU admission, 30-day return to the emergency department, and the number of outpatient follow-ups.

## 2. Materials and Methods

### 2.1. Study Population

Our study population included all adult patients who underwent pulmonary resection for lung cancer at a single hospital from 1 January 2016 to 31 December 2020. This hospital is part of a network that encompasses multiple hospitals within a large, integrated healthcare system, serving over 4.9 million individuals in Northern California. All operations were performed by six thoracic surgeons within our integrated regional thoracic surgery network, which comprised thoracic patients from 12 medical centers in the surrounding area of Northern California. The start date was chosen based on the year (2014) that our health system initially regionalized thoracic surgery care and gave a 2-year roll-out period to be bypassed in hopes of mitigating any of the learning curve for the regionalization process [9,10].

#### 2.1.1. Study Design

This study was a retrospective cohort utilizing convenience sampling. Data were extracted from existing clinical and administrative databases (namely the patient electronic medical record (EMR) in which data are collected as part of routine clinical care). Any additional research databases used that use the EMR and the cancer registry as source data undergo quality assurance by analysts who resolve any data inconsistencies when merging data from different administrative/clinical sources. Within this study itself, any inconsistent data or outliers were adjudicated by physician chart review. Patients were identified by diagnosis of lung cancer and thoracic surgery procedure codes. Collected patient characteristics included age, sex, race/ethnicity, neighborhood deprivation index, body mass index, Charlson Comorbidity Index scores, primary language, insurance type, home region, lung cancer stage, systemic therapy, surgical approach (open, VATS, or robot), history of alcohol use disorder, smoking status, and extent of surgical resection (pneumonectomy, lobectomy, segmentectomy, or wedge). Charlson Comorbidity Index scores were computed using ICD-10 codes extracted from EMR data and validated via routine system audits. Race/ethnicity was self-reported at time of registration and aligned with US census categories. Smoking-related data were also obtained from the patient electronic medical record, which provided self-reported information about smoking habits closest to and before the date of surgery. Neighborhood deprivation index scores were based on residential addresses and were calculated based on eight census variables in the domains of income/poverty, education, employment, housing, and occupation with patients classified into quartiles (with quartile 1 being the least deprived and quartile 4 being the most deprived) [11]. Home regions were broken into local, North, West, South, Northeastern, and Southeastern. The home region was included with the aim of accounting for potential unmeasured differences in delivery of care such as travel distance. Outcomes collected included time from cancer diagnosis to surgery, intensive care unit admission, the length of stay, return to the emergency department within 30 days, readmission within 30 days, number of surgical and non-surgical outpatient appointments within 30 and 90 days, overall mortality within 30 and 90 days, recurrence within one year and two years, and survival at one year and at two years. The cancer diagnosis date in our cohort was from the regional cancer registry, which adheres to the Surveillance, Epidemiology, and End Results Program. This was either a clinical or histopathological diagnosis of cancer. The LOS was calculated from the time of hospital admission for the pre-operative management to the time of discharge. Surgical outpatient appointments were defined as follow-up visits with the thoracic surgical team for post-operative care. Non-surgical outpatient appointments included visits with primary care physicians or pulmonologists and specialists other than thoracic surgeons. These visits were tracked starting from the date of surgery. Outcomes were compared by race/ethnicity.

#### 2.1.2. Statistical Analysis

Categorical sociodemographic and clinical characteristics as well as surgical and cancer outcomes were compared between race/ethnicity groups using both the Chi-square and Fisher’s exact tests. Normally distributed continuous variables such as age were compared between race/ethnicity groups using Student’s *t*-test. Non-normally distributed continuous variables (such as the number of outpatient and virtual appointments, length of stay, and time from diagnosis to surgery) were compared using Kruskal–Wallis tests. Multivariable regression models were used to assess for independent variable associations with outcomes of interest. In cases of continuous outcomes of interest, linear regression models were performed. Both the unadjusted (OR) and the adjusted odds ratios (aOR) with 95% confidence intervals were used to report results for logistic regression models, and both the unadjusted (Beta) and the adjusted betas (aBeta) with 95% confidence intervals were used to report results for linear regression models. Multivariable models incorporated variables of age, sex, body mass index, type of thoracic surgery, neighborhood deprivation index, insurance status, cancer stage, Charlson Comorbidity Index scores, and receipt of adjuvant chemotherapy. Our study was powered on the hypothesis that Asian patients will have 20% 30-day ED readmission compared with 5% of non-Asian patients. Using a two-sided continuity corrected Chi-square test with a 5% significance level, our a priori power and sample size calculations have 82% power to detect the difference between a proportion of 20% for Asian race/ethnicity and 5% for non-Asian race/ethnicity when the sample sizes are 50 and 400, respectively. Our final sample size exceeds 450. Analyses were performed using SAS 9.4 (Cary, NC, USA). A *p*-value of <0.05 was considered statistically significant. ORs were used for binary outcomes (e.g., ED return), while β-coefficients were used for continuous outcomes (e.g., the length of stay). Normality was assessed using Shapiro–Wilk tests, and non-parametric methods (e.g., Kruskal–Wallis) were used where appropriate.

## 3. Results

Overall, 645 patients were included in the study. Table 1 displays basic demographic and clinical characteristics of the patient population. Of the 645 included patients, 18.6% were Asian (n = 120), 9.6% were Black (n = 62), 8.8% were Hispanic (n = 57), and 2.8% were of other ethnic/racial background (n = 18). A majority of the cohort, 64.3%, were female (n = 415), and 35.7% were male (n = 230). Non-Hispanic White patients tended to be older and live in the least deprived quartile neighborhoods (Table 1). Among each race/ethnicity, the percentage of patients insured by Medicaid was highest among Asian patients (Table 1). Patients underwent one of three types of thoracic surgery: open (8.1%), robotic (3.9%), or video-assisted (88.1%).

### Thoracic Surgery Outcomes

Table 2 summarizes outcomes after lung cancer resection by race/ethnicity. There was no statistically significant difference by race/ethnicity for 30-day readmission to hospital, 30-day return to the ED, ICU transfer, and 30- and 90-day overall mortality. No significant differences were observed in the number of all and non-surgical outpatient appointments within 30 and 90 days after surgery. There was a statistically significant difference (*p* = 0.04) in the number of surgical outpatient visits within 30 days between race/ethnicities with those of Hispanic and Other race/ethnicity having a median visit of 1 (IQR: 0–1) and 1 (IQR: 1–2), respectively. All other race/ethnicity groups had a median visit of 0 (IQR: 0–1). Once the follow-up period was extended to 90 days after surgery, there was no significant difference observed in the number of surgical outpatient appointments. Multivariable analysis showed Asian patients had significantly fewer surgical outpatient visits (β −0.17; 95% CI (−0.33, −0.02)) compared with other race/ethnicities (Table 3). While this could be due to sociocultural differences that were not directly measured, this association requires further investigation and should not be interpreted as causal. We also found no statistically significant differences in cancer outcomes such as recurrence, overall mortality, and time from diagnosis to surgery between groups (Table 2). It is worth noting that our study defines time to surgery as referenced in our previous study by Banks et al., who, in their study “Association of Surgical Timing with Outcomes in Early Stage Lung Cancer”, define time to surgery as the interval between either most recent suspicious CT/PET imaging or histopathological diagnosis of cancer to the date of surgery, with 58% of patients undergoing surgery between 5 and 12 weeks [12]. The recurrence was confirmed based on histopathological evidence of the specimen.

In bivariate analyses, we also observed a significant difference (*p* = 0.04) in the length of hospital stay as well, with the longest hospital stay in hours being noted in the Black race/ethnicity (Median: 56.3 (IQR: 32.4–86.1)) and the shortest hospital stay being noted in other ethnicities (Median: 45.8 (IQR: 32.1–80.4)), closely followed by Hispanic patients (Median: 47 (IQR: 32.1–57.7)) (Table 2).

In the bivariate analysis presented in Table 2, there was no significant difference in the 30-day return to the ED outcome among different race/ethnicities. Table 4 summarizes the adjusted odds ratios (aORs) and 95% confidence intervals (CIs) for factors associated with the 30-day return to the emergency department (ED) outcome. Multivariable analysis revealed that patients with Medicaid insurance had significantly higher odds of 30-day ED return (aOR 3.29; 95% CI 1.26–8.59), while Asian patients experienced significantly lower odds of 30-day ED return compared with non-Hispanic White patients (aOR 0.51; 95% CI 0.27–0.98). Other variables, including age, gender, BMI, and Charlson Comorbidity Index scores, were not significantly associated with 30-day ED return.

## 4. Discussion

Research on disparities among lung cancer patients primarily focuses on the differences in treatment modalities and their associated outcomes. While understanding these aspects is crucial, there is a lack of sufficient data on patients’ interactions within a particular healthcare system after their treatment. Our study is novel in that it utilizes a racially and ethnically diverse integrated health system with no loss of follow-up to study its dynamic relationship with patients. Understanding these disparities is crucial because the care of patients with serious illnesses like lung cancer goes beyond obvious clinical outcomes, such as appropriate treatment administration or overall mortality. Furthermore, it involves their ongoing interactions with the healthcare system, including readmissions, visits to the emergency department (ED), and outpatient visits.

Our multivariable analysis highlights disparities in 30-day ED return rates, with Medicaid insurance significantly associated with higher odds of ED utilization. This finding aligns with prior research suggesting that socioeconomic factors, including insurance type, play a critical role in healthcare utilization patterns. Additionally, Asian patients had lower odds of 30-day ED return, a finding that may reflect differences in healthcare-seeking behaviors or potential unmeasured factors such as social support or care navigation. These results underscore the importance of addressing social determinants of health to reduce disparities in post-surgical care. One explanation for this finding could be that Asian patients have better post-treatment outcomes than other ethnic groups [13]. However, we did not otherwise find differences in clinical outcomes between other different racial/ethnic groups. It is possible that cultural beliefs about health and the role of healthcare providers differ among different racial/ethnic populations [14,15]. While some individuals may seek professional help at the first sign of complications, others may reserve visits to the emergency department (ED) for more severe symptoms. However, the fact that the readmission rate was not significantly higher among Asian patients, which would be expected if their ED presentations were for more severe medical complications, makes this explanation less plausible. It is worth noting that the Asian American population in Northern California is highly diverse, with various subgroups having distinct cultural, socioeconomic, and healthcare experiences. Understanding this diversity is essential for interpreting healthcare behaviors and outcomes. Future research should aim to disaggregate data to reflect the distinct experiences of different Asian subgroups. Finally, it is well established that access to healthcare influences visits to the emergency department. In 2002, the Institute of Medicine published a report on remarkable ethnic/racial disparities in the US healthcare delivery system [16]. Specifically, it was observed that minorities were more inclined to use the emergency department (ED) as their entry point into the healthcare system. They were also more likely to be uninsured and less likely to have a primary care provider [17]. Perhaps concordantly, our analysis revealed that patients with Medicaid insurance visited the ED more frequently. However, in our integrated health system, this should not be due to restricted access to Medicaid providers. The reason for increased ED visits among patients receiving Medicaid in our system is not entirely clear; however, plausible suggestions (in addition to increased cultural and language barriers) could be farther distance from regionalized thoracic centers within our health system.

It is worth noting that 64% of our studied cohort were female patients. This higher proportion of female patients could be influenced by several factors, including gender-specific trends in lung cancer incidence and outcomes as well as institution-specific patient demographics. Although men have historically been more likely to be diagnosed with lung cancer, recent trends show that the incidence of lung cancer among women is increasing. As shown in our recent study, our population consists heavily of female patients and a higher proportion of Asian American female non-smoking patients due to the large demographic population in Northern California; thus, this may partially attribute to the higher female cohort status in our study [18]. Additionally, local referral patterns and demographic characteristics of our center could also play a role. Understanding these factors is essential for interpreting our study results, and further research could provide additional insights into gender-specific outcomes in thoracic surgery.

Interestingly, within our cohort, Asian patients make up most Medicaid patients. This paradox is worth exploring. At the national level, Asians constitute 24.3% of Medicaid recipients [19]. While Medicaid patients in general might be more likely to seek care in the ED due to socioeconomic factors such as lack of transportation, limited availability of Medicaid providers or appointments, Asians might exhibit different healthcare-seeking behavior. It is also possible that Asian patients experience more language barriers in healthcare settings [20]. Future research should specifically analyze the interaction between race and insurance status to provide a more granular analysis of post-operative healthcare utilization. This would involve collecting detailed data on insurance types and examining their impact within different racial/ethnic groups.

The finding that Black patients had a statistically significant longer hospital stay compared with other races/ethnicities is interesting as well. The LOS is a recognized metric for healthcare efficiency. It has significant clinical and financial implications for patients and hospitals. It reflects differences in post-operative care and resource utilization. In a retrospective cohort study involving more than 1 million patients with both surgical and medical diagnosis, Ghosh et al. found that wealthier patients had a shorter LOS and that Black patients had a significantly longer LOS compared with White patients [21]. While the type of surgery performed and pre-existing comorbidities certainly influence the LOS, we adjusted for these variables with multivariable regression models in our analysis that incorporated variables such as age, sex, type of thoracic surgery, BMI, and others. Even though the difference was less than a day and is unlikely to be clinically significant, financial implications are affected by every hour of extended inpatient stay [22]. Health disparities, including chronic comorbidities among Black patients, could contribute to a prolonged hospital stay. Our multivariable analyses attempted to address this confounding characteristic via the inclusion of Charlson Comorbidity Index scores as a covariate in our models. Furthermore, a range of socioeconomic factors could present barriers to accessing timely healthcare, potentially leading to a longer hospital stay. Although our analysis controlled for factors such as neighborhood deprivation index scores, insurance status, and geographic location, there are other variables that could be considered, including transportation availability.

While integrated systems reduce structural barriers such as access to healthcare and insurance issues, our findings still show there is room for improvement, as we saw that individual-level differences (e.g., socioeconomic status, cultural beliefs) can still contribute to disparities in utilization. This is particularly interesting in the context of multiple prior studies that have found such major differences among patients undergoing lung cancer treatment [19,20,21,22,23,24,25,26]. The absence of disparities in our study is likely attributable to the nature of our integrated healthcare system, where patients have insurance coverage and access to primary care, reducing barriers to healthcare access and ensuring more uniform care delivery. Likewise, the process of regionalization further reduces healthcare disparities by creating networks of hospitals that collaborate, share resources, and standardize care by developing uniform protocols for pre-operative assessments, surgical procedures, post-operative monitoring, and patient education [27,28]. We also have advanced care providers on our thoracic teams that are solely dedicated to maintaining outpatient follow-up, ensuring prompt scheduling, and reducing loss to follow-up. Our hospital system utilizes MyChart, an electronic health record platform which allows for communication between patients and their providers. During the pre-operative period, patients can access MyChart to receive instructions and access educational resources related to their procedure. Post-operatively, MyChart is used to manage follow-up care. Patients can access their appointments, message their care team members, or access post-discharge care instructions. Care team-initiated virtual and/or in-person post-operative visits are utilized as needed to check on patients’ progress or to follow up on any concerns that were not resolved during in-person follow-up. This approach aimed to provide equitable care and might have contributed to the lack of observed racial disparities in post-operative outcomes. However, as noted in this study, outcome disparities can still exist in such a system. This emphasizes even further the need for all institutions to track outcomes at a more granular level than is typically reported. Ultimately, outcome disparities may vary by institution, and targeted solutions will likely vary as well.

Our study found an 18% rate of returning to the ED. According to the current literature, the 30-day return to the ED rates for post-operative patients can vary widely depending on the type of surgery, patient population, and healthcare setting. Studies have reported rates ranging as high as 10% to 20% for various surgical procedures [29,30,31]. Since our cohort consists of patients who underwent lung resection—a complex thoracic procedure—it is not surprising that the 30-day return is as high. The high return rate underscores the importance of investigating potential disparities in post-operative care and outcomes. Identifying and addressing factors contributing to higher ED returns can help improve care and reduce unnecessary ED visits.

The primary limitation of our retrospective study is that we cannot assume a causal relationship between patient characteristics and outcomes. Additionally, in a non-randomized setting, our ability to control confounding variables was limited. The smoking status of our patients was not analyzed as a variable in assessing health outcome disparities following lung resection. This decision was primarily due to inconsistencies in both provider documentation and the recording of patients’ social histories, which compromised the validity of the available data. We acknowledge this as a limitation and recommend that future studies collect standardized behavioral data. Furthermore, our study does not include data on perioperative morbidity, which could certainly influence potential outcome disparities. Another key limitation of our study is that the Asian population in our cohort had higher Medicaid coverage compared with national trends, where Medicaid coverage among Asians is generally lower. This discrepancy suggests that our cohort may not fully represent the broader Asian population or national trends in Medicaid coverage. As a result, our findings may not be generalizable to all Asian individuals in the general population. It is worth noting that our study did not include lung cancer biomarker data. Such markers can vary by race/ethnicity and could significantly influence treatment approaches and outcomes in lung cancer. Future studies should include more diverse cohorts to better reflect national patterns and enhance the generalizability of the findings.

Our findings regarding Medicaid utilization are specific to the US healthcare context. In countries with universal healthcare systems, disparities associated with insurance status may differ significantly. For example, insurance coverage in the US differs from that in Europe and other regions of the world, which may limit the generalizability of our findings to other countries. These findings reflect disparities within the US healthcare system and may not be directly generalizable to other countries. Future studies should explore whether similar patterns exist in other healthcare contexts.

Findings from an integrated healthcare system like Kaiser Permanente may not generalize to broader US populations, especially those receiving care in fragmented or under-resourced systems. However, our most recent paper on the representativeness of lung cancer patients in Kaiser Permanente was actually more diverse and applicable to the general population compared with National Cancer Institute-Designated Cancer Centers. Therefore, while there are limitations to our health system, the diverse heterogeneity in race/ethnicity in our health system mitigates systemic biases. However, our study highlights the potential benefits of integrated healthcare systems. By providing insurance coverage and ensuring access to primary care, these systems can create a more equitable healthcare environment where patients receive timely and appropriate care regardless of their racial or ethnic background. Primary care providers play a key role in early diagnosis, ongoing management of comorbidities, and coordination of care, which can significantly impact perioperative and cancer outcomes. Furthermore, the standardization of care protocols within our integrated health system may have contributed to the uniformity in outcomes across different racial and ethnic groups. Standardized protocols ensure that all patients receive consistent and evidence-based care, reducing variability in treatment and outcomes. Other healthcare systems could benefit from implementing similar standardized care pathways to minimize disparities. Finally, healthcare systems should incorporate strategies to address social determinants of health, such as socioeconomic status, education, and housing. By understanding and mitigating these factors, hospitals can offer more comprehensive and equitable care.

## 5. Conclusions

Racial and ethnic disparities in lung cancer outcomes are well documented. The unique aspect of our study is that it is conducted within an integrated health system. This can explain relative parity by race/ethnicity. It is a system where primary care physicians, oncologists, thoracic surgeons, and radiologists are all part of a single system with quick and open communication. In addition, our patients have quick access to their providers and healthcare records, appointments, etc., via secure messaging and an online portal. Patient and care coordinators and schedulers help ensure that patients attend their appointments, which has led to very low rates of loss to follow-up at less than 3% annually. We did find significant racial/ethnic differences in the LOS and 30-day return to the ED within our health system that should be evaluated at a more granular level to identify process improvement protocols. Different racial/ethnic groups experienced different rates in the length of stay and of return to the ED within 30 days, suggesting that we can focus on better patient education in language- and culture-specific materials in order to help mitigate these disparities. The efforts to recognize and target these disparities are necessary for equitable patient care.

## Figures and Tables

**Table 1:** Demographic and clinical characteristics of adult lung cancer resection patients 2016–2020 by race/ethnicity (n = 645).

Patient Characteristic	Total n = 645 (100%)	Asian n = 120 (18.6%)	Black n = 62 (9.6%)	Hispanic n = 57 (8.8%)	Other n = 18 (2.8%)	Non-Hispanic White n = 388 (60.2%)	Chi Square *p*-Value
Age at Index, Mean (SD)	68.4 (9.8)	66.5 (9.8)	67.3 (8.8)	66.8 (11.4)	66.0 (10.9)	69.6 (9.5)	0.01 ^a^
Sex							
Female	415 (64.3)	78 (65.0)	39 (62.9)	36 (63.2)	11 (61.1)	251 (64.7)	0.99
Male	230 (35.7)	42 (35.0)	23 (37.1)	21 (36.8)	7 (38.9)	137 (35.3)	
BMI							
Normal (<25)	279 (43.3)	77 (64.2)	24 (38.7)	19 (33.3)	10 (55.6)	149 (38.4)	<0.01
Overweight (25–29.9)	206 (31.9)	32 (26.7)	22 (35.5)	21 (36.8)	7 (38.9)	124 (32.0)	
Obese (>30)	160 (24.8)	11 (9.2)	16 (25.8)	17 (29.8)	1 (5.6)	115 (29.6)	
CCI							
Score 0–2	103 (16.0)	22 (18.3)	5 (8.1)	9 (15.8)	4 (22.2)	63 (16.2)	0.01
Score 3–4	270 (41.9)	57 (47.5)	18 (29.0)	27 (47.4)	3 (16.7)	165 (42.5)	
Score 5–6	120 (18.6)	17 (14.2)	13 (21.0)	7 (12.3)	4 (27.8)	78 (20.1)	
Score 7+	152 (23.6)	24 (20.0)	26 (41.9)	14 (24.6)	6 (33.3)	82 (21.1)	
Type of Thoracic Surgery							
Open	52 (8.1)	9 (7.5)	5 (8.1)	4 (7.0)	3 (16.7)	31 (8.0)	<0.01
Robotic	25 (3.9)	2 (1.7)	2 (3.2)	3 (5.3)	0 (0.0)	18 (4.6)	
Video-Assisted	568 (88.1)	109 (90.8)	55 (88.7)	50 (87.7)	15 (83.3)	339 (87.4)	
NDI							
Q1 (least deprived)	212 (32.9)	39 (32.5)	5 (8.1)	8 (14.0)	9 (50.0)	151 (38.9)	<0.01
Q2	209 (32.4)	35 (29.2)	15 (24.2)	15 (26.3)	4 (27.8)	139 (35.8)	
Q3	135 (20.9)	31 (25.8)	14 (22.6)	22 (38.6)	2 (11.1)	66 (17.0)	
Q4 (most deprived)	89 (13.8)	15 (12.5)	28 (45.2)	12 (21.1)	2 (11.1)	32 (8.3)	
Language							
Asian	35 (5.4)	34 (28.3)	0 (0.0)	0 (0.0)	1 (5.6)	0 (0.0)	<0.01 ^b^
English	591 (91.6)	85 (70.8)	62 (100.0)	39 (68.4)	17 (94.4)	388 (100.0)	
Other	1 (0.2)	1 (0.8)	0 (0.0)	0 (0.0)	0 (0.0)	0 (0.0)	
Spanish	18 (2.8)	0 (0.0)	18 (31.6)	18 (31.6)	0 (0.0)	0 (0.0)	
Medicaid							
Yes	22 (3.4)	8 (6.7)	3 (4.8)	3 (5.3)	1 (5.6)	7 (1.8)	0.09 ^b^
No	620 (96.6)	111 (93.3)	59 (95.2)	54 (94.7)	17 (94.4)	379 (98.2)	
Patient Home Region							
Diablo Napa	214 (34.8)	28 (24.4)	15 (24.6)	17 (31.5)	4 (25.0)	150 (40.7)	0.75 ^b^
East Bay	210 (34.2)	52 (45.2)	39 (63.9)	25 (46.3)	4 (25.0)	90 (24.4)	
Fresno Central	9 (1.5)	0 (0.0)	0 (0.0)	1 (1.9)	0 (0.0)	8 (2.2)	
Sac Valley	4 (0.7)	2 (1.7)	0 (0.0)	0 (0.0)	0 (0.0)	2 (0.5)	
Santa Clara	1 (0.2)	0 (0.0)	0 (0.0)	0 (0.0)	0 (0.0)	1 (0.3)	
West Bay	177 (28.8)	33 (28.7)	7 (11.5)	11 (20.4)	8 (50.0)	118 (32.0)	
Type of Resection							
Wedge	144 (22.3)	17 (14.2)	17 (27.4)	13 (22.8)	2 (11.1)	95 (24.5)	0.5 ^b^
Segmentectomy	40 (6.2)	6 (5.0)	3 (4.8)	3 (5.3)	2 (11.1)	26 (6.7)	
Lobectomy	450 (69.8)	95 (79.2)	41 (66.1)	40 (70.2)	13 (72.2)	261 (67.3)	
Bilobectomy/Pneumonectomy	11 (1.7)	2 (1.7)	1 (1.6)	1 (1.8)	1 (5.6)	6 (1.6)	
Cancer Stage							
I	395 (61.2)	73 (60.8)	37 (59.7)	28 (49.1)	9 (50.0)	248 (63.9)	0.09 ^b^
II	109 (16.9)	22 (18.3)	9 (14.5)	12 (21.1)	2 (11.1)	64 (16.5)	
III	107 (16.6)	19 (15.8)	15 (24.2)	14 (24.6)	7 (38.9)	52 (13.4)	
IV	34 (5.3)	6 (5.0)	1 (1.6)	3 (5.3)	0 (0.0)	24 (6.2)	
Systemic Therapy							
Neoadjuvant chemo	7 (1.1)	2 (1.7)	0 (0.0)	1 (1.8)	0 (0.0)	4 (1.0)	0.83 ^b^
Adjuvant chemo	165 (25.6)	34 (28.3)	18 (29.0)	15 (26.3)	6 (33.3)	92 (23.7)	0.71
Neoadjuvant radiation	3 (0.5)	0 (0.0)	1 (1.6)	0 (0.0)	0 (0.0)	2 (0.5)	0.61 ^b^
Alcohol Use Disorder	26 (4.0)	3 (2.5)	1 (1.6)	3 (5.3)	0 (0.0)	19 (4.9)	0.62 ^b^
Smoking Status							
Current	90 (14.0)	11 (9.2)	14 (22.6)	5 (8.8)	1 (5.6)	59 (15.2)	<0.01
Former	267 (41.4)	17 (14.2)	28 (45.2)	25 (43.9)	8 (44.4)	189 (48.7)	
Never	288 (44.7)	92 (76.7)	20 (32.3)	27 (47.4)	9 (50.0)	140 (36.1)	

^a^ *p*-value calculated using Student’s *t*-test; ^b^ *p*-value calculated using Fisher’s exact test.

**Table 2:** Surgical and cancer outcomes after lung cancer resection by race/ethnicity (n = 645).

Surgical Outcome	Total n = 645 (100%)	Asian n = 120 (18.6%)	Black n = 62 (9.6%)	Hispanic n = 57 (8.8%)	Other n = 18 (2.8%)	Non-Hispanic White n = 388 (60.2%)	Chi-Square *p*-Value
30-Day Readmission to Hospital, n (%)							
Yes	59 (9.2)	11 (9.2)	4 (6.5)	3 (5.3)	2 (11.1)	39 (10.1)	0.73
No	586 (90.9)	109 (90.8)	58 (93.6)	54 (94.7)	16 (88.9)	349 (90.0)	
30-Day Return to ED, n (%)							
Yes	113 (17.5)	14 (11.7)	9 (14.5)	10 (17.5)	2 (11.1)	78 (20.1)	0.24
No	532 (82.5)	106 (88.3)	53 (85.5)	47 (82.5)	16 (88.9)	310 (79.9)	
ICU Transfer, n (%)							
Yes	24 (3.7)	6 (5.0)	3 (4.8)	2 (3.5)	0 (0.0)	13 (3.4)	0.81
No	621 (96.3)	114 (95.0)	59 (95.2)	55 (96.5)	18 (100.0)	375 (96.7)	
30-Day Overall Mortality, n (%)							
Yes	5 (0.8)	1 (0.8)	0 (0.0)	1 (1.8)	0 (0.0)	3 (0.8)	0.85
No	640 (99.2)	119 (99.2)	62 (100.0)	56 (98.3)	18 (100.0)	385 (99.2)	
90-Day Overall Mortality, n (%)							
Yes	12 (1.9)	1 (0.8)	1 (1.6)	2 (3.5)	0 (0.0)	8 (2.1)	0.74
No	633 (98.1)	119 (99.2)	61 (98.4)	55 (96.5)	18 (100.0)	380 (97.9)	
Number of Outpatient Appointments within 30 days, Median (IQR)	2 (1–3)	2 (0.5–3)	1.5 (1–3)	2 (1–3)	3 (2–5)	2 (1–3)	0.07 ^a^
Number of Surgical Outpatient Appointments within 30 days, Median (IQR)	0 (0–1)	0 (0–1)	0 (0–1)	0 (0–1)	1 (0–1)	0 (0–1)	0.12 ^a^
Number of Non-surgical Outpatient Appointments within 30 days, Median (IQR)	1 (0–2)	1 (0–2)	1 (0–2)	1 (0–3)	2 (1–4)	1 (0–2)	0.23 ^a^
Number of Virtual Appointments within 30 days, Median (IQR)	4 (2–6)	3 (2–6)	4 (2–6)	4 (3–6)	3 (2–6)	4 (2–5)	0.72
Number of Outpatient Appointments within 90 days, Median (IQR)	4 (2–8)	3 (1–7.5)	4 (2–7)	5 (2–9)	7 (3–14)	4 (2–8)	0.22 ^a^
Number of Surgical Outpatient Appointments within 90 days, Median (IQR)	0 (0–1)	0 (0–1)	0 (0–1)	1 (0–1)	1 (1–2)	0 (0–1)	0.04 ^a^
Number of Non-surgical Outpatient Appointments within 90 days, Median (IQR)	3 (1–8)	3 (1–7)	3 (1–7)	4 (2–9)	4 (2–10)	3 (1–7.5)	0.44 ^a^
Number of Virtual Appointments within 90 days, Median (IQR)	5 (3–9)	5 (3–8)	6 (3–12)	6 (3.5–8.5)	4.5 (3–12)	5 (3–9)	0.26
Length of Stay (hours), Median (IQR)	49.8 (31.3–73.0)	50.7 (31.6–78.7)	56.3 (32.4–86.1)	47.0 (32.1–57.7)	45.8 (32.1–80.4)	47.3 (31.2–58.8)	0.04 ^a^
Cancer Outcome							
Time from Cancer Diagnosis to Surgery in days, Median (IQR)	38 (13–62)	36.5 (20–57.5)	43 (20–72)	38 (28–61)	34.5 (13–50)	38 (0–65.5)	0.7 ^a^
Recurrence within one year, n (%)							
Yes	31 (4.8)	7 (5.8)	4 (6.5)	1 (1.8)	2 (11.1)	17 (4.4)	0.48
No	614 (95.2)	113 (94.2)	58 (93.6)	56 (98.3)	16 (88.9)	371 (95.6)	
Recurrence within two years, n (%)							
Yes	73 (11.3)	17 (14.2)	7 (11.3)	5 (8.8)	3 (16.7)	41 (10.6)	0.72
No	572 (88.7)	103 (84.8)	55 (88.7)	52 (91.2)	15 (83.3)	347 (89.4)	
One-Year Overall Mortality, n (%)							
Yes	36 (5.6)	5 (4.2)	5 (8.1)	4 (7.0)	1 (5.6)	21 (5.4)	0.84
No	609 (94.4)	115 (95.8)	57 (91.9)	53 (93.0)	17 (94.4)	367 (94.6)	
Two-Year Overall Mortality, n (%)							
Yes	69 (10.7)	10 (8.3)	8 (12.9)	6 (10.5)	3 (16.7)	42 (10.8)	0.8
No	576 (89.3)	110 (91.7)	54 (87.1)	51 (89.5)	15 (83.3)	346 (89.2)	

^a^ *p*-value calculated using Kruskal–Wallis test.

**Table 3:** Adjusted odds ratios (aORs) and beta estimates of surgical and cancer outcomes after lung cancer resection by race/ethnicity (n = 645).

	Race/Ethnicity (Ref = Non-Hispanic White) Unadjusted Odds Ratios and Betas			
Patient Outcome	Asian	Black	Hispanic	Other
30-day Return to ED [OR (95% CI)]	0.53 (0.29, 0.97)	0.68 (0.32, 1.43)	0.85 (0.41, 1.75)	0.50 (0.11, 2.21)
30-day Readmission to Hospital [OR (95% CI)]	0.90 (0.45, 1.82)	0.62 (0.21, 1.79)	0.50 (0.15, 1.67)	1.12 (0.25, 5.05)
Recurrence within two years [OR (95% CI)]	1.40 (0.76, 2.56)	1.08 (0.46, 2.52)	0.81 (0.31, 2.15)	1.69 (0.47, 6.10)
Length of Stay [Beta (95% CI)]	17.18 (−5.23, 39.59)	39.33 (9.99, 68.67)	−6.72 (−37.15, 23.71)	18.8 (−32.92, 70.53)
All Outpatient Visits within 30 days [Beta (95% CI)]	−0.30 (−0.83, 0.23)	−0.23 (−0.93, 0.46)	0.51 (−0.21, 1.23)	1.20 (−0.03, 2.43)
All Outpatient Visits within 90 days [Beta (95% CI)]	0.05 (−1.59, 1.69)	0.74 (−1.41, 2.89)	1.53 (−0.70, 3.75)	2.20 (−1.59, 5.98)
Non-surgical Outpatient Visits within 30 days [Beta (95% CI)]	−0.22 (−0.72, 0.28)	−0.30 (−0.95, 0.36)	0.61 (−0.07, 1.28)	0.70 (−0.45, 1.85)
Non-surgical Outpatient Visits within 90 days [Beta (95% CI)]	0.07 (−1.54, 1.67)	0.68 (−1.42, 2.78)	1.54 (−0.63, 3.72)	0.92 (−2.78, 4.62)
Surgical Outpatient Visits within 30 days [Beta (95% CI)]	−0.08 (−0.23, 0.08)	0.06 (−0.14, 0.26)	−0.10, (−0.31, 0.11)	0.50 (0.14, 0.85)
Surgical Outpatient Visits within 90 days [Beta (95% CI)]	−0.02 (−0.23, 0.19)	0.06 (−0.22, 0.33)	−0.02 (−0.30, 0.27)	1.28 (0.79, 1.76)
	Race/Ethnicity (ref = Non-Hispanic White) Adjusted Odds Ratios and Betas			
Patient Outcome	Asian	Black	Hispanic	Other
30-day Return to ED [aOR (95% CI)]	0.51 (0.27, 0.98)	0.61 (0.27, 1.36)	0.88 (0.41, 1.91)	0.45 (0.10, 2.10)
30-day Readmission to Hospital [aOR (95% CI)]	0.97 (0.45, 2.06)	0.61 (0.20, 1.87)	0.48 (0.14, 1.68)	1.28 (0.28, 6.03)
Recurrence within two years [aOR (95% CI)]	1.40 (0.73, 2.69)	0.91 (0.37, 2.27)	0.73 (0.27, 2.02)	1.19 (0.31, 4.52)
Length of Stay [Beta (95% CI)]	15.47 (−7.22, 38.17)	33.72 (4.21, 63.23)	−10.03 (−40.33, 20.27)	10.74 (−39.72, 61.20)
All Outpatient Visits within 30 days [aBeta (95% CI)]	−0.44 (−0.97, 0.08)	−0.35 (−1.02, 0.33)	0.43 (−0.27, 1.12)	0.66 (−0.50, 1.82)
All Outpatient Visits within 90 days [aBeta (95% CI)]	−0.51 (−1.94, 0.93)	−0.19 (−2.06, 1.67)	1.04 (−0.87, 2.96)	0.40 (−2.80, 3.58)
Non-surgical Outpatient Visits within 30 days [aBeta (95% CI)]	−0.27 (−0.76, 0.22)	−0.42 (−1.05, 0.22)	0.52 (−0.14, 1.17)	0.30 (−0.79, 1.39)
Non-surgical Outpatient Visits within 90 days [aBeta (95% CI)]	−0.33 (−1.73, 1.06)	−0.26 (−2.08, 1.56)	1.05 (−0.82, 2.91)	−0.69 (−3.80, 2.41)
Surgical Outpatient Visits within 30 days [aBeta (95% CI)]	−0.17 (−0.33, −0.02)	0.07 (−0.13, 0.27)	−0.09 (−0.30, 0.12)	0.36 (0.01, 0.71)
Surgical Outpatient Visits within 90 days [aBeta (95% CI)]	−0.17 (−0.39, 0.04)	0.06 (−0.21, 0.34)	0.00 (−0.29, 0.28)	1.09 (0.62, 1.56)

Notes: All reported adjusted odds ratios (aORs) and adjusted beta (aBeta) estimates are adjusted for age, sex, BMI, type of thoracic surgery, neighborhood deprivation index, Medicaid status, cancer stage, Charlson Comorbidity Index scores, and receipt of adjuvant chemotherapy.

**Table 4:** Adjusted odds ratios and 95% CIs of 30-day return to ED (n = 645).

Characteristic	Adjusted OR	95% CI
Age	1.00	0.98–1.03
Race/Ethnicity		
Asian	0.51	0.27–0.98
Black	0.61	0.27–1.36
Hispanic	0.88	0.41–1.91
Other	0.45	0.10–2.10
White	ref	ref
Gender		
Male	0.89	0.57–1.39
Female	ref	ref
BMI		
Normal	ref	ref
Overweight	0.99	0.61–1.63
Obese	0.82	0.47–1.42
Charlson Comorbidity Index		
Score 0–3	ref	ref
Score 4+	2.42	1.39–4.20
Type of Thoracic Surgery		
Open	1.21	0.56–2.60
Robotic/Video-Assisted	ref	ref
NDI		
Q1 (least deprived)/Q2	1.05	0.65–1.68
Q3/Q4 (more deprived)	ref	ref
Medicaid		
Yes	3.29	1.26–8.59
No	ref	ref
Cancer Stage		
I	ref	ref
II	1.51	0.82–2.80
III	1.17	0.57–2.40
IV	1.54	0.58–4.06
Adjuvant Chemotherapy	0.73	0.39–1.37

## Data Availability

Data supporting the reported results are not publicly available due to privacy and ethical restrictions. The data were obtained from an integrated health system and contain sensitive patient information, which cannot be shared publicly. Access to the data may be available upon reasonable request and with permission from the institution’s ethics committee.
